# Enhancement of instrumented ultrasonic tracking images using deep learning

**DOI:** 10.1007/s11548-022-02728-7

**Published:** 2022-09-03

**Authors:** Efthymios Maneas, Andreas Hauptmann, Erwin J. Alles, Wenfeng Xia, Sacha Noimark, Anna L. David, Simon Arridge, Adrien E. Desjardins

**Affiliations:** 1 Wellcome/EPSRC Centre for Interventional and Surgical Sciences, University College London, London, W1W 7TY UK; 2 Department of Medical Physics and Biomedical Engineering, University College London, London, WC1E 6BT UK; 3 Department of Computer Science, University College London, London, WC1E 6BT UK; 4 Research Unit of Mathematical Sciences, University of Oulu, FI-90014 Oulu, Finland; 5 School of Biomedical Engineering and Imaging Sciences, King’s College London, London, SE1 7EH UK; 6 Institute for Women’s Health, University College London, London, WC1E 6HX UK; 7 NIHR UCLH Biomedical Research Centre, London, W1T 7DN UK

**Keywords:** Deep learning, Interventional devices, Ultrasonic needle tracking

## Abstract

**Purpose:**

Instrumented ultrasonic tracking provides needle localisation during ultrasound-guided minimally invasive percutaneous procedures. Here, a post-processing framework based on a convolutional neural network (CNN) is proposed to improve the spatial resolution of ultrasonic tracking images.

**Methods:**

The custom ultrasonic tracking system comprised a needle with an integrated fibre-optic ultrasound (US) transmitter and a clinical US probe for receiving those transmissions and for acquiring B-mode US images. For post-processing of tracking images reconstructed from the received fibre-optic US transmissions, a recently-developed framework based on ResNet architecture, trained with a purely synthetic dataset, was employed. A preliminary evaluation of this framework was performed with data acquired from needle insertions in the heart of a fetal sheep *in vivo*. The axial and lateral spatial resolution of the tracking images were used as performance metrics of the trained network.

**Results:**

Application of the CNN yielded improvements in the spatial resolution of the tracking images. In three needle insertions, in which the tip depth ranged from 23.9 to 38.4 mm, the lateral resolution improved from 2.11 to 1.58 mm, and the axial resolution improved from 1.29 to 0.46 mm.

**Conclusion:**

The results provide strong indications of the potential of CNNs to improve the spatial resolution of ultrasonic tracking images and thereby to increase the accuracy of needle tip localisation. These improvements could have broad applicability and impact across multiple clinical fields, which could lead to improvements in procedural efficiency and reductions in risk of complications.

## Introduction

Ultrasound-guided needle insertions are widely performed in many clinical contexts [1]. A key challenge in these procedures is to accurately identify the needle tip relative to the ultrasound imaging plane. Instrumented ultrasonic tracking has recently been shown to be a promising solution. This method involves ultrasonic communication between an ultrasound imaging probe and a needle [2–5]. Communication can be effected in reception-mode, with a receiver integrated into the needle, or in transmission-mode, with an integrated ultrasound transmitter. These reception/transmission modes include corresponding transmissions/receptions from individual elements of the imaging probe, and image reconstruction to obtain tracking images. The acquisition of tracking images can be interleaved with B-mode ultrasound images for real-time operation [6].

With ultrasonic tracking, the resolution at which the needle tip can be resolved is of critical importance. Recently, a framework based on a convolutional neural network (CNN) was proposed to enhance the image quality of instrumented reception-mode ultrasonic tracking images that were acquired with ultrasound reception from the needle tip [7]. Here, we use the principle of time-reversal to motivate the use of this network for transmission-mode ultrasonic tracking. Specifically, we hypothesise that this CNN-based framework, which was trained purely on synthetic reception-mode tracking data, can improve the spatial resolution of *in vivo* transmission-mode tracking images. Additionally, we investigate the impact on CNN performance by comparing different parameters with which to generate ground truth images for CNN training. This preliminary evaluation was performed using data acquired from a preclinical fetal sheep model.

## Methods

**Fig. 1:**
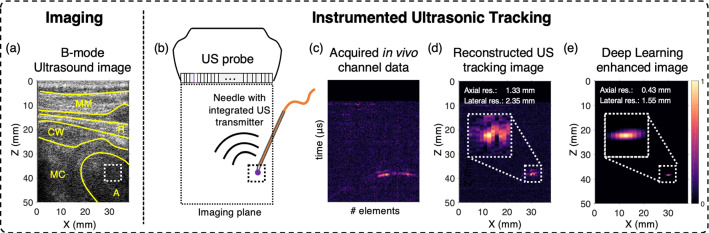
Ultrasonic tracking with a needle inserted in the heart of a fetal sheep *in vivo*. In this frame, the needle tip is located at a depth of 38.4 mm. With B-mode ultrasound (US) imaging (**a**), identification of the needle tip (blue dot) was challenging. The transmission-mode implementation of ultrasonic tracking is shown schematically (**b**), with a fibre-optic ultrasound transmitter integrated within a needle, and with parallel reception of these transmissions by all elements of the clinical US imaging probe. Reconstruction of the received channel data (**c**) yielded the ultrasonic tracking image (**d**). Using the trained network, the image quality of tracking images was enhanced (**e**); the axial resolution with which the needle tip could be visualised improved from 1.33 to 0.43 mm and the lateral resolution improved from 2.36 to 1.55 mm. MM: myometrium, R: rib, CW: chest wall, MC: myocardium, A: atrium

The instrumented ultrasonic tracking system [6] comprised two components: a needle with an integrated fibre-optic ultrasound transmitter and a clinical ultrasound imaging system (SonixMDP, Ultrasonix Medical Corporation, Richmond, BC, Canada). The ultrasound transmitter was fabricated with a custom polydimethylsiloxane-carbon nanotube composite coating applied to the distal end of an optical fibre [8]. Ultrasound generation was achieved with pulsed light excitation of this coating via the photoacoustic effect. The ultrasound system was operated in research mode, which allowed for interleaved acquisitions of B-mode ultrasound images (Fig. 1a) and tracking images, with the latter obtained from simultaneous reception from all 128 transducer elements of the imaging probe (SonixDAQ, Ultrasonix Medical Corporation, Richmond, BC, Canada). The received A-line signals (Fig. 1b) were reconstructed offline (sound speed: 1500 m/s) with a Fourier domain method implemented in k-wave [9] to form the ultrasonic tracking image (Fig. 1c).

Post-processing of tracking images was performed using a framework [7] based on a modified ResNet architecture [10, 11], which was trained using synthetic data. Generation of these images involved simulating transmitted ultrasound fields with a fast near field method (FOCUS) [12] and then applying a Fourier domain method for image reconstruction [9]. A representative output from the network is shown in Fig. 1d. With simulations, the tissue medium was assumed to be homogeneous with an absence of attenuation and a uniform sound speed of 1500 m/s. The ultrasound probe was modelled as a set of 128 rectangular planar transducers, distributed equidistantly across an aperture of 38.4 mm. The synthetic training dataset comprised 1000 images that included a single point source, which was varied in position across the axial and lateral dimensions of the imaging plane. Zero-mean Gaussian noise was added to the channel data, which resulted in SNR values that ranged from 10.5 to 56.1. After reconstruction, envelope detection via the Hilbert transform followed by scaling was performed to restrict the solution space to the range [0,1] for faster convergence during training. For each tracking image, a ground truth image was generated with a single point source corresponding to the ultrasound transmitter location, convolved with a Gaussian kernel. Three kernels with various sizes ($\sigma = \big [\sigma _{Z}, \sigma _{X}\big ]= \big [1, 1\big ], \big [4, 2\big ], \big [8, 4\big ]$ pixels, where *Z* and *X* are the axial and lateral dimensions, respectively) were used to train three CNNs and their performance was compared. The choice of anisotropic kernels elongated in the axial dimension was made to compensate for the difference in axial and lateral sampling rates in the channel data.

The CNN framework comprised a modified residual neural network with 16 residual blocks [7]. Each block consisted of 2 convolutional layers with 64 channels width and 3$\times$3 convolutional kernels and biases, with a rectified linear unit as nonlinearity between the 2 convolutional layers. To achieve faster convergence during training, patches of 64$\times$64 pixels were used in batches of 16, which were extracted randomly from the training set. The CNN was implemented with TensorFlow v1.13 and Keras v2.2.4, on a workstation comprising an Nvidia 1080Ti GPU. Training was performed for 80 epochs with a synthetic validation set (generated in the same way as the training set) by minimising the L1-loss between the reconstructed and ground truth images (ADAM optimizer; initial step size: 0.001).

To evaluate the performance of the trained network, three needle insertions into the heart of a fetal sheep were performed *in vivo*, as part of a broader set of preclinical experiments. The procedure was conducted in accordance with the U.K. Home Office regulations and the Guidance for the Operation of Animals (Scientific Procedures) Act (1986). Ethics approval was provided by the joint animal studies committee of the Royal Veterinary College and University College London (UCL), U.K.

Reconstructed tracking images were evaluated in terms of the axial and lateral spatial resolution of the needle tip. For resolution measurements, a $5\times 5$ mm bounding box centred around the maximum amplitude of the needle tip was used; the maximum intensity projections of the axial and lateral profiles were obtained to calculate the corresponding full-width-half-maximum (FWHM) values.

## Results & Discussion

An example of a needle insertion is shown in Fig. 1. Across all insertions, the needle tip ranged in depth from 23.9 to 38.4 mm. Signals from the ultrasound transmitter were clearly apparent in A-lines from approximately half of the transducer elements (Fig. 1c); it appeared that these signals originated solely from the needle tip. Application of the trained CNN to tracking images yielded average spatial resolution improvements from 2.11 to 1.58 mm in the lateral dimension and 1.29 to 0.46 mm in the axial dimension, respectively. Additionally, background noise suppression was observed, which was flattened to near constant values when the network is applied.

**Fig. 2:**
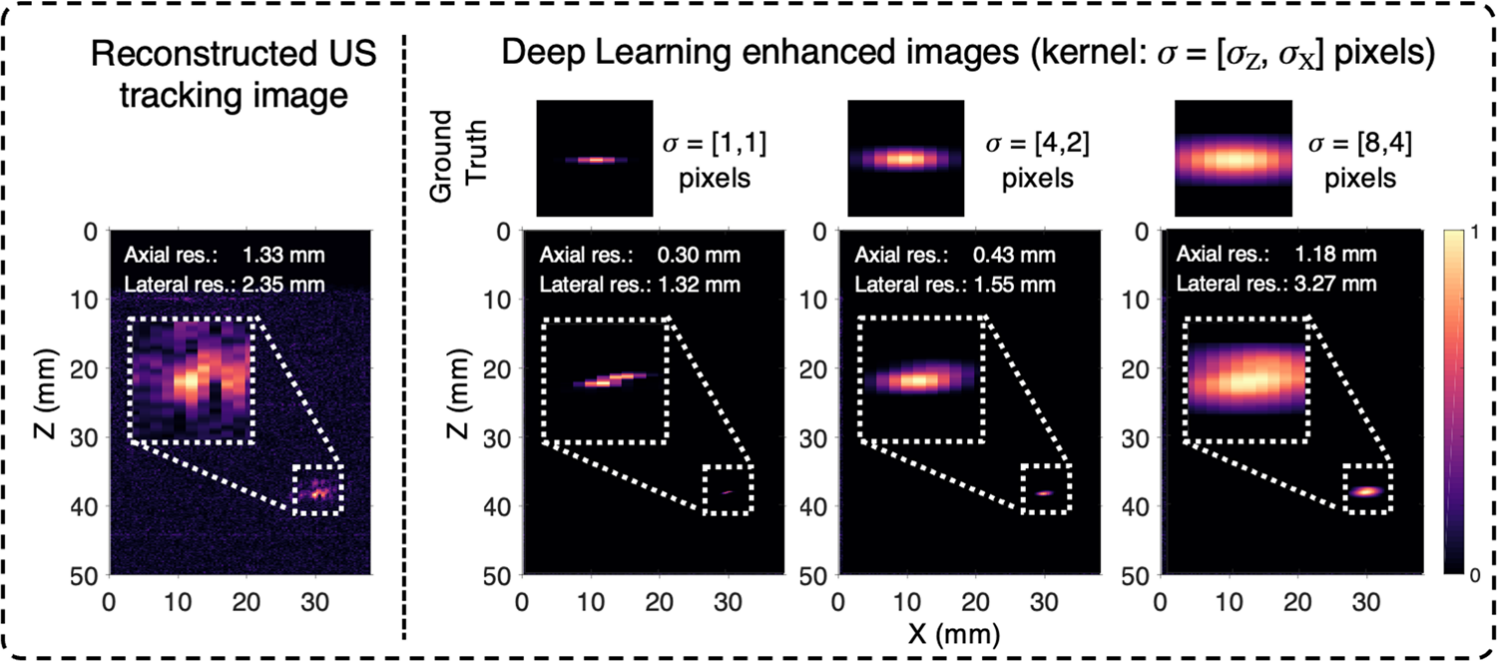
Impact of the Gaussian kernel dimensions on the performance of the trained CNN. The raw channel dataset shown in Fig. 1 was used here as an exemplar. Three CNNs were trained separately with ground truth images that were generated with a single point source convolved with a kernel of various dimensions: $\sigma = \big [\sigma _{Z}, \sigma _{X}\big ]= \big [1, 1\big ], \big [4, 2\big ], \big [8, 4\big ]$ pixels, where *Z* and *X* are the axial and lateral dimensions, respectively. Using a small Gaussian kernel ($\sigma = [1,1]$ pixels) can lead to the generation of multiple objects in the enhanced tracking image, as obtained in this example. A kernel with $\sigma = [4,2]$ pixels yielded improved performance, with fewer instances of multiple objects in the enhanced tracking image (a single object was obtained in this example). Further increasing the axial and lateral dimensions of the kernel ($\sigma = [8,4]$ pixels) tended to decrease the resolution with which the needle tip was visualised in the enhanced tracking image

Additionally, the impact of the dimensions of the Gaussian kernel used to obtain ground truth images was investigated (Fig. 2). The use of a single pixel kernel ($\sigma = [1,1]$ pixels) appears to be sub-optimal as it often led to multiple objects in the tracking image. Conversely, using a larger kernel ($\sigma = [8,4]$ pixels) tended to increase the size of the needle tip object in the enhanced tracking image, thereby decreasing the spatial resolution. Accordingly, an intermediate-sized kernel with $\sigma = [4,2]$ pixels was chosen, which led to resolution improvements in both the axial and lateral dimensions.

In ultrasonic tracking, obtaining large training sets for training neural networks accurate estimation of ground truth is often challenging [1]. Manual annotation is typically time-intensive and can also introduce further uncertainty when there is low visibility of the needle tip. In this context, the use of synthetic data for training CNNs is attractive. The success with a purely synthetic training dataset in this study stemmed in part from the simplicity of the acquisition scheme combined with a highly accurate numerical model for tracking images. Notably, these images comprised only one point-like object, namely the ultrasound transmitter. By comparison, synthesising B-mode ultrasound images of tissue comprising a multitude of objects, such as those encountered during needle insertions, gives rise to many challenges owing to the much greater dimensionality of this problem [13, 14].

Several topics could be addressed in order to improve upon the results presented here. First, further optimisation of the dimensions of the kernels used to generate ground truth images could be performed. This might lead to the use of kernel dimensions that vary with the ground truth needle tip position, perhaps mirroring spatial variations in the point spread function of needle tips in reconstructed US tracking images. Second, a Kalman filtering approach could be used to improve needle tip position estimates by incorporating data from multiple ultrasonic tracking frames [15], thereby acknowledging continuity of the needle path through tissue. Third, variations in the sound speed and acoustic attenuation of the imaged medium could be included to the training dataset with a view to improve robustness for different tissue structures. These variations were present in the *in vivo* dataset, which the framework handled well. Fourth, explorations of the performance of the CNN with far fewer simultaneously-received A-lines used to generate the tracking images would be of interest in terms of limiting system complexity and cost. Finally, in order to remove the image reconstruction step, end-to-end network approaches that use A-lines acquired directly from the ultrasound imaging probe as inputs [16, 17] could be investigated in this context.

## Conclusion

This study demonstrated for the first time that a CNN-based framework trained only on synthetic data can be used to improve the spatial resolution of transmission-mode ultrasonic tracking images acquired *in vivo*. These results provide strong indications of the potential to improve the spatial resolution in ultrasonic tracking and thereby to increase the accuracy with which the needle tip is localised, with the aim of improving the efficiency and safety of ultrasound-guided needle insertions.
